# Prevalence and factors associated with risky sexual behaviors among adolescent girls and young women in Rwanda: evidence from a national population-based cross-sectional HIV impact assessment, 2018-2019

**DOI:** 10.11604/pamj.2026.54.12.49800

**Published:** 2026-05-15

**Authors:** Ange Joseline Iradukunda, Basile Ikuzo, Claude Mambo Muvunyi, Sara Winterhalter, Zephanie Nzeyimana

**Affiliations:** 1 James Lind Institute, Geneva, Switzerland, USA; 2 Rwanda Biomedical Centre, Kigali, Rwanda, USA; 3 New York City Metropolitan Area, Faye, USA

**Keywords:** Risky sexual behaviors, adolescent girls and young women, HIV/AIDS, unintended pregnancy

## Abstract

**Introduction:**

globally, adolescent girls and young women (AGYW) aged 15 to 24 face higher risks of acquiring HIV and unintended pregnancies, primarily due to risky sexual behaviors (RSBs) such as engaging in sex for money and having sex without a condom. This study assessed factors associated with RSBs among AGYW in Rwanda.

**Methods:**

this study analysed weighted data from the 2019 Rwanda Population-based HIV Impact Assessment (RPHIA), a cross-sectional study that collected data from October 2018 to March 2019. The analysis used the Jackknife method in survey logistic regression. Statistical significance was assumed for a p-value less than 0.05.

**Results:**

among 6,753 adolescent girls and young women aged 15-24 years (mean age: 20.9 years), 44.1% reported ever having sex, corresponding to 2,980 participants. Among sexually active AGYW, the weighted prevalence of risky sexual behaviors was 43.9%. The most common RSBs include sex without a reliable method of contraception (24.8%), extramarital sex without a condom (13.5%), sex before the age of 15 (11.5%), sex for gifts/favors/money (9.9%), having multiple sexual partners (9.1%), transgenerational sex (1.7%), and anal sex (0.8%). Factors determining RSBs among the AGYW who reported ever having sex include having ever been pregnant (aOR: 4.3 with 95% C.I: 1.4-13.4), being pregnant at the time of the interview (aOR: 9.5 with 95% C.I: 6.8-13.4), having sex before the age of 15 (aOR: 1.8 with 95% C.I: 1.1-2.9), age at first sex ranging between 15-17 years (aOR: 1.3 with 95% C.I: 1.0-1.7), and living in a household with one or more children aged below 15 years (aORs ranging from 1.6 to 1.9).

**Conclusion:**

overall, RSBs remain a public health threat among young females in Rwanda, putting them at an elevated risk of HIV infection and unintended pregnancies. Thus, there is a need for sexual and reproductive health services appropriate for AGYW, focusing on delaying the age of sexual debut, promoting condom use, abstinence, and modern contraception among sexually active young females.

## Introduction

Risky sexual behaviors (RSBs) are practices that increase the likelihood of unplanned pregnancies and sexually transmitted infections (STIs), including HIV/AIDS. These behaviors encompass engaging in sex before marriage, having multiple sexual partners, having sex without a condom, having sex without a reliable modern contraception for birth control, having sex under the influence of alcohol, and engaging in transactional sex [1-3]. Besides unintended pregnancies and sexually transmitted infections, risky sexual behaviors result in lifelong health and economic problems for young females and their families [4]. Among adolescent girls and young women (AGYW), the prevalence of RSBs remains disproportionately higher than it does among their male counterparts and older women, exacerbating their vulnerability to HIV and unintended pregnancies [5]. Globally, AGYW account for approximately 42% of all new HIV infections, with 80% of these cases occurring in sub-Saharan Africa [6]. Additionally, 38% of all pregnancies worldwide are unintended [7]. This is equivalent to approximately 4,000 new HIV infections among AGYW each week in 2023, with 3,100 of these infections occurring in sub-Saharan Africa [6]. Various studies in sub-Saharan Africa have associated these risks with the high prevalence of RSBs among youth, and regional variations are influenced by socioeconomic, cultural, and educational factors.

Studies across sub-Saharan Africa highlight the high prevalence of RSBs among youth, with notable regional variations. In Ethiopia, 43.1% of youth engaged in RSBs, with lower rates among those attending youth centres [7]. In Zambia, 9.5% initiated sexual activity before age 15, and 61.2% had unprotected extramarital sex [8]. Similarly, AGYW in eastern Africa face heightened risks. In Tanzania, studies reported that up to 80% of youths-initiated sex by age 18 [9]. In Kenya, the determined, resilient, empowered, AIDS-free, mentored and safe partnership project found that 74% of sexually active AGYW did not use condoms, with key risk factors including cohabitation, being out of school, and living away from parents [10]. Some studies have reported factors or determinants of RSBs among this vulnerable group in low and middle-income countries. In Rwanda, findings from the PHIA provide critical insights into the extent of RSBs among AGYW. According to reported descriptive statistics of the survey, 51.7% of AGYW reported engaging in sex without a condom, 10.0% had two or more sexual partners, and 5.7% initiated sexual activity before the age of 15. Additionally, 83.7% of adolescent girls aged 15-19 engaged in sexual activity with non-marital, non-cohabiting partners in the past year, with only 42.6% using condoms. Among young women aged 20-24 years, 48.4% reported extramarital sexual activity, with only 34.0% using condoms [11]. These alarming statistics emphasise the need to understand the determinants of RSBs for targeted interventions to mitigate the risks associated with RSBs in this demographic.

While there are limited studies on the determinants of RSBs using population-based data, some studies have reported contributing factors in low- and middle-income countries. In many countries, women still rely on men's income. This dependency, particularly in situations of poverty, leads some women to engage in transactional sex for basic needs, a luxurious lifestyle, or as a means to express their love to men [12]. Additionally, limited access to education, insufficient employment opportunities, inadequate awareness of sexual and reproductive health, and deeply rooted cultural norms further exacerbate engagement in RSBs [13]. Young females aged 15-24 in Rwanda continue to bear a disproportionate burden of new infections and unintended pregnancies, largely due to RSBs, despite significant progress in HIV prevention. It is essential to understand the specific factors influencing these behaviors to optimise health resource allocation and implement effective prevention strategies. This study aimed to determine the prevalence of risky sexual behaviors and their associated factors among adolescent girls and young women in Rwanda using population-based data.

## Methods

**Study design:** the study analysed data from the Rwanda PHIA survey, a cross-sectional study, which collected data from Oct 2018 to March 2019. The PHIA project started in 2014 with the overall aim of establishing the national status of the HIV epidemic. PHIA projects are conducted by countries' ministry of health under the funding of the U.S. President's Emergency Plan for AIDS Relief (PEPFAR) through the U.S. Centres for Disease Control and Prevention (CDC) with technical support from ICAP at Columbia University and MGIC at the University of Maryland, Baltimore. The projects collect population-based information on the HIV epidemic and the characteristics of the study participants, including their demographic, health, reproductive, and behavioral information [14].

Rwanda PHIA used a two-stage, stratified cluster sampling design based on the 2012 census in Rwanda. In the first stage, 375 enumeration areas were selected using probability proportional to size, stratified by province. In the second stage, households (HHs) were randomly chosen within each enumeration area, with an average of 30 households per enumeration area. The sample size was designed to ensure representative national estimates of HIV incidence among adults (15-49 years) with a relative standard error (RSE) of ≤ 38% and provincial estimates of viral load suppression (VLS) with confidence intervals (CIs) of ±10%. A subnational HIV incidence estimate for Kigali City required sample reallocation while maintaining precision for VLS in other provinces. Young adolescents in selected HHs were also surveyed to estimate national HIV prevalence with an RSE of ≤ 28% for a 0.3% prevalence or ≤ 34% for a 0.2% prevalence. The final target sample sizes were 24,523 adults and 5,411 young adolescents for the entire Rwanda PHIA Survey. Within households, all people found in the households were enrolled in the survey [11].

**Setting:** the 2018/2019 Rwanda Population-Based HIV Impact Assessment was conducted in Rwanda, a landlocked country in eastern Africa. Renowned for its picturesque hilly terrain, Rwanda is often referred to as the “Land of a Thousand Hills”.

**Sample size:** this study analysed PHIA data of 6,753 adolescent girls and young women aged between 15 and 24 years who were enrolled in the 2018/2019 Rwanda PHIA. However, factors associated with risky sexual behaviors were determined only among AGYW who have ever engaged in sexual intercourse.

**Participants:** this study was of females aged between 15 and 24 years, referred to as adolescent girls and young women throughout this manuscript, who were enrolled in the 2018/2019 PHIA survey in Rwanda. The study excluded females who did not report ever having sex by the time of data collection. Of the 6,753 enrolled in the PHIA Survey, 2980 reported ever having sex.

**Variables:** a data analysis plan was developed to guide data analysis and address the current research questions. Independent variables included demographic characteristics, type of health coverage, sources of information like television and radio, sexual behaviors, and obstetric characteristics of the study participants. Independent variables were selected based on known literature about risky sexual behaviors and their availability in PHIA datasets. For example, early initiation of sexual activity has been shown to elevate the likelihood of having multiple sexual partners, experiencing sexual abuse, and engaging in transactional sex (15). The dependent variable was defined as engaging in at least one risky sexual behavior, such as having sex without a condom, initiating sexual activity before the age of 15, engaging in sex without a reliable method of contraception, engaging in transgenerational sex (with a partner at least 10 years older), engaging in transactional sex, and other behaviors as reported in Table 1 and Table 1.1.

**Table 1:** characteristics of AGYW, population-based HIV impact assessment in Rwanda (October 2018 to March 2019)

Variable	Frequency (n)	Percent (%)
Age, mean (Min-Max)	20 (15-24)	-
15-19 years	970	32.5
20-24 years	2,010	67.5
Province	-	-
Kigali city	695	23.3
South	475	15.9
West	692	23.2
North	569	19.1
East	549	18.4
Rural/Urban	-	-
Urban	919	30.8
Rural	2,061	69.2
Marital status	-	-
Married	230	7.7
Living together	639	21.4
Divorced/Separated/widowed	168	5.6
Single	1,943	65.2
Have you ever been married?	-	-
Yes	1,038	34.8
No	1,942	65.2
Economic category	-	-
I-Lowest	478	16.0
II-Second	548	18.4
III-Middle	517	17.4
IV-Fourth	519	17.4
V-Highest	918	30.8
Education	-	-
No education	77	2.6
Primary	1,636	54.9
Secondary	1,200	40.3
More than Secondary	67	2.3
I received cash from my occupation in the last 12 months	-	-
Yes	1,076	36.1
No	1,904	63.9
Household head gender	-	-
Male	1,908	64.0
Female	1,072	36.0
Whether household members have health insurance	-	-
Yes	2,480	83.2
No	500	16.8
Sexual behaviors	-	-
Age at first sex	-	-
Not known	418	14.0
Below 15	343	11.5
15-17	733	24.6
18-24	1,486	49.9
Engaging in sexual activity before the age of 15	-	-
Yes	2,637	88.5

**Table 1.1:** characteristics of AGYW, population-based HIV impact assessment in Rwanda (October 2018 to March 2019)

Variable	Frequency (n)	Percent (%)
**Whether household members have health insurance**		
Yes	2,480	83.2
No	500	16.8
**Sexual behaviors**		
**Age at first sex**		
Not known	418	14.0
Below 15	343	11.5
15-17	733	24.6
18-24	1,486	49.9
**Engaging in sexual activity before the age of 15**		
Yes	2,637	88.5
No	343	11.5
**Engaged in a sexual relationship with a person with whom she resides**		
No	1,757	68.3
Yes	814	31.7
**Age of the male sexual partner**		
14 to 30	1,708	85.7
31 to 40	246	12.4
41-50	38	1.9
**Engaged in a sexual relationship with a person with whom she resides**	1,757	68.3
No	814	31.7
Yes		
**Obstetric information**		
**Frequency of pregnancy**		
Never/unknown	1,479	49.6
Once	1,073	36.0
Two or more times	428	14.4
**Whether currently pregnant**		
Yes	289	9.7
No	2,691	90.3
**Whether ever given birth**		
Yes	1,268	83.8
No	246	16.3
**Number of Children**		
None	139	11.0
1 Child	1,122	88.5
2 to 4	7	0.6
**Total**	**2,980**	**100**

**Bias:** PHIA surveys employ advanced sampling and study design techniques that account for regional and country-level variability and probabilities. To ensure regional sample variability is accurately reflected, we used the Jackknife method in survey logistic regression.

**Quantitative variables:** including age and number of children, were grouped to make them categorical. Participants were aged between 15 and 24 years. Age was grouped into two categories based on the adolescent period, while the number of children was grouped into three categories: no children, 1 to 3 (the nationally recommended number of children per woman of reproductive age), and four or more for those with a high number of children.

**Statistical methods:** data were analysed at the univariate level to describe participants' characteristics and determine the prevalence of risky sexual behaviors among the study population. Factors associated with risky sexual behaviors were determined among AGYW who reported ever having sex. Bivariate analysis was conducted by cross-tabulating participants' characteristics with risky sexual behaviors using the chi-square test to assess statistical significance. Finally, the Jackknife variance estimation technique was applied in survey logistic regression to account for survey design complexities used in PHIA surveys, which include stratification, clustering of the sample, and unequal weighting. It was also selected to minimise bias and ensure robust statistical results. This analysis generated adjusted odds ratios for all variables that were statistically associated with risky sexual behaviors at the bivariate level.

**Ethics approval and consent to participate in the study:** per the Declaration of Helsinki, the Rwanda Population-based HIV Impact Assessment (RPHIA) obtained ethical clearance from the Rwanda National Ethics Committee. The dataset does not include personal identifiers of human subjects enrolled in Rwanda PHIA. We officially obtained the RPHIA dataset from ICAP at Columbia University upon request. Data analysis of the RPHIA data was conducted for research purposes only, with the overall goal of informing targeted services to prevent new HIV infections and unintended pregnancies in Rwanda, especially among AGYW.

## Results

This section presents the findings of the study, covering the demographic characteristics of the study participants, risky sexual behaviors, and associated factors among AGYW who participated in the 2018/2019 Rwanda PHIA.

**Study participants:** of the 6,753 adolescent girls and young women (AGYW) aged 15 to 24 years enrolled in the RPHIA, 2,980 (44.1%) reported having ever had sex, and their mean age was 20.6 years. Of the AGYW who reported ever having sex, the majority were aged 15 to 19 years (65.5%), from rural areas (69.2%), single (65.2%), had primary education (54.9%), and lived in households with health insurance (83.2%). Considering the sexual and obstetric information of the participants, 49.6% had never been pregnant, 9.7% were pregnant at the time of data collection, and 16.3% had ever given birth. Of those who had ever given birth, 88.5% had one child. Further information is found in Table 1 and Table 1.1.

**Figure 1:**
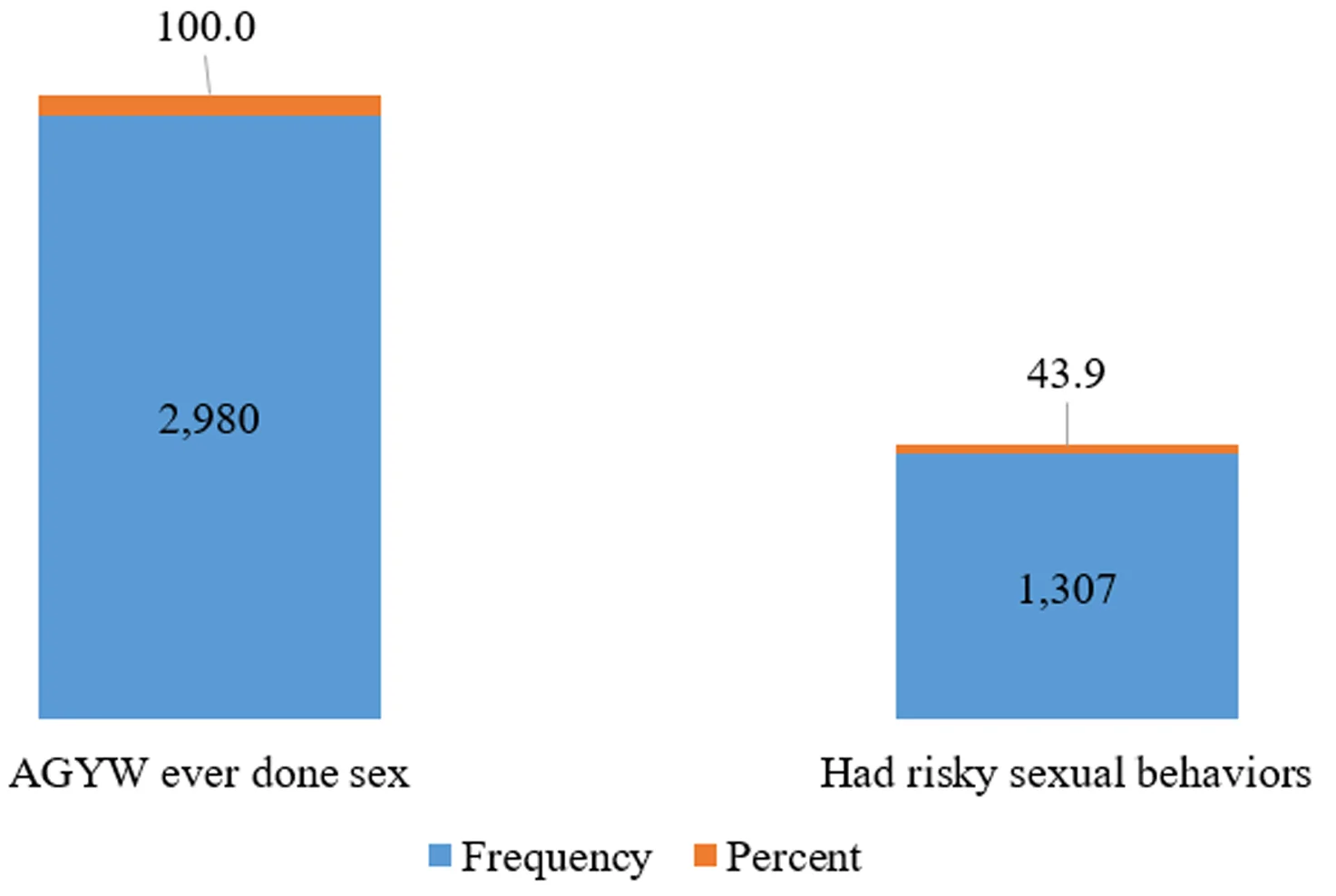
prevalence of risky sexual behaviors among AGYW ever engaged in sexual intercourse, population-based HIV impact assessment in Rwanda (October 2018 to March 2019)

**The prevalence of risky sexual behaviors among AGYW who have ever engaged in sexual intercourse:** this study shows that the prevalence of risky sexual behaviors was 43.9% among the 2,980 AGYW who reported ever having sex. Figure 1 illustrates further information.

**Figure 2:**
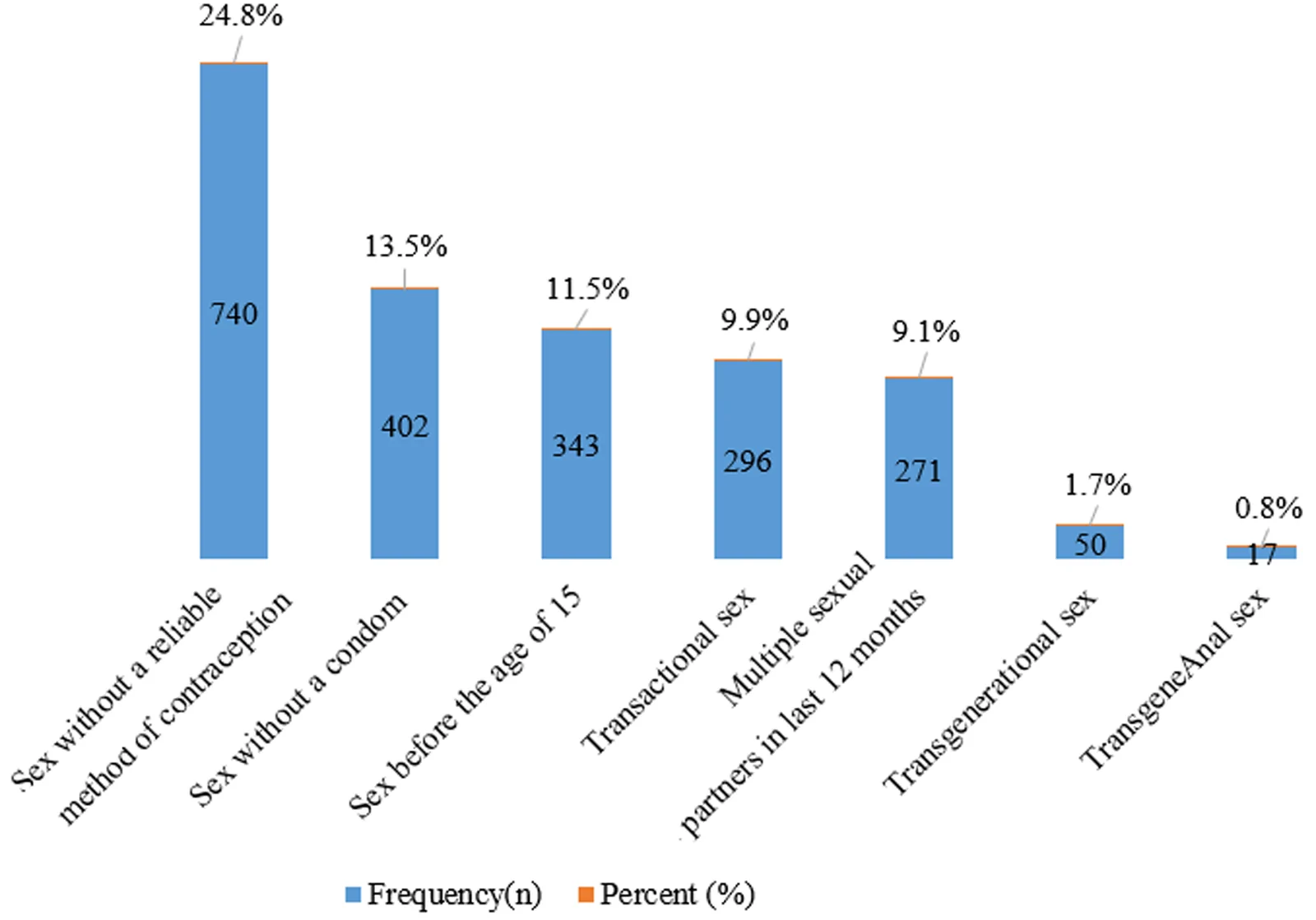
types of risky sexual behaviors identified among adolescent girls and young women who reported ever having sex, population-based HIV impact assessment in Rwanda (October 2018 to March 2019)

**Types of risky sexual behaviors identified among AGYW:** to assess RSBs, the study is based on respondents' answers to RSB questions like transactional sex (defined as sex for money, favor, or gifts), transgenerational sex (having sex with someone 10 years older than she is), and sex without a reliable method (sex while not using a modern contraception method), among others. Various RSBs among AGYW who reported ever having sex were identified, including having sex without a reliable method of contraception (24.6%), sex without a condom (13.5%), sex before the age of 15 (11.5%), transactional sex (9.9%), having multiple sexual partners (9.1%), transgenerational sex (1.7%), and anal sex (0.8%). Figure 2 provides further details.

**Factors associated with risky sexual behaviors among AGYW who reported ever having sex and were enrolled in the Rwanda PHIA survey:** this part presents the association between RSBs and participants' characteristics, including demographic, sexual, obstetric, and household characteristics. Significantly higher prevalence rates of having RSBs were found among AGYW aged 20-24 years (50.3%), those with a divorced/separated/widowed marital status (55.2%), those ever married (2.3%), those separated (divorced/widowed) (56.6%), those ever married (55.2%), households having 1 to 3 children (47.5%), those engaged in transactional sex (100%), those who experienced their first sex at age between 15-17 years (59.8%), those who had sex before age 15 years (45.8%), those who had sex with someone they live with (53.8%), those who have ever been pregnant (58.4%), those not pregnant at the time of the data collection interview (46.8%), and those who have ever given birth (64.8%). Table 2 and Table 2.2 show participants' characteristics associated with risky sexual behaviors among AGYW aged 15 to 24 years who reported ever having sex, while the strength and direction of the association are shown in Table 3.

**Table 2:** association between participants' characteristics and having risky sexual behaviors among AGYW, population-based HIV impact assessment in Rwanda (October 2018 to March 2019)

Participants' characteristics	Risky sexual behaviors No Yes		n	P Value
Age	-	-	-	-
15-19 years	675(69.6)	295(30.4)	970	<0.001
20-24 years	998(49.7)	1,012(50.)	2,010	
Province	-	-	-	-
Kigali city	374(53.8)	321(46.2)	695	0.05
South	276(58.1)	199(41.9)	475	-
West	418(60.4)	274(39.6)	692	-
North	304(53.4)	265(46.6)	569	-
East	301(54.8)	248(45.2)	549	-
Rural/Urban	-	-	-	-
Urban	493(53.7)	426(46.4)	919	0.067
Rural	1,180(57.3)	881(42.7)	2,061	
Have you ever been married to a man or lived with him as if you were married?	-	-	-	-
Yes	465(44.8)	573(55.2)	1,038	<0.001
No	1,208(62.2)	734(37.8)	1,942	
Economic category	-	-	-	-
I-Lowest	288(60.3)	190(39.7)	478	0.4
II-Second	306(55.8)	242(44.2)	548	
III-Middle	287(55.5)	230(44.5)	517	
IV-Fourth	284(54.7)	235(45.3)	519	
V-Highest	508(55.3)	410(44.7)	918	
Education				
No education	44(57.1)	33(42.9)	77	0.446
Primary	898(54.9)	738(45.1)	1,636	
Secondary	690(57.5)	510(42.5)	1,200	
More than Secondary	41(61.2)	26(38.8)	67	
Whether payment was received, either in money or goods, for work done in the last twelve months	-	-	-	-
Yes	582(54.1)	494	1,076	0.09
No/Not known	1,091(57.3)	813(42.7)	1,904	
Household characteristics	-	-	-	-
The number of children in the household				
No child	256(64.7)	140(35.4)	396	<0.001
1 to 3	965(52.5)	874(47.5)	1,839	-
4 to 6	408(61.2)	259(38.8)	667	-
7 to 11	44(56.4)	34(43.6)	78	-
Possessing any type of health insurance	-	-	-	-
Yes	1,404(56.6)	1,076(43.)	2,480	0.248
No	269(53.8)	231(46.2)	500	-
Household head gender	-	-	-	-
Male	1,072(56.2)	836(43.8)	1,908	0.949

**Table 2.2:** association between participants' characteristics and having risky sexual behaviors among AGYW, population-based HIV impact assessment in Rwanda (October 2018 to March 2019)

Participant’s characteristics	Risky sexual behaviors No Yes		n	P -Value
Female	601(56.1)	471(43.9)	1,072	-
**Whether or not the household has a radio**	**-**	-	-	-
Yes	848(55.1)	691(44.9)	1,539	0.237
No	825(57.3)	616(42.8)	1,441	-
Whether a household owns a television	**-**	-	-	-
Yes	365(58.1)	263(41.9)	628	0.26
No	1,308(55.6)	1,044(44.4)	2,352	-
**Sexual and obstetric characteristics of the study participants**	**-**	-	-	-
**Age at first sex**	**-**	-	-	**-**
Not known	415(99.3)	3(0.7)	418	**<0.001**
Below 15	243(70.9)	100(29.1)	343	**-**
15-17	295(40.2)	438(59.8)	733	**-**
18-24	720(48.5)	766(51.5)	1,486	**-**
**Sex before the age of 15**	**-**	-	-	**-**
Yes	1430(54.2)	1207(45.8)	2,637	**<0.001**
No	243(70.8)	100(29.2)	343	
**Engaged in a sexual relationship with a person she resides with**	**-**	-	-	**-**
No	1296(59.9)	868(40.1)	2,164	**<0.001**
Yes	377(46.2)	439(53.8)	816	-
**Age of the male partner**	**-**	-	-	-
14 to 30	639(37.4)	1,068(62.6)	1,707	0.544
31 to 40	101(41.1)	145(58.9)	246	-
41 to 50	14(36.8)	24(63.2)	38	-
**Have you ever been pregnant?**	**-**	-	-	-
No	1048(70.9)	431(29.1)	1,479	**<0.001**
Yes	625(41.6)	876(58.4)	1,501	**-**
**Whether the person is currently pregnant**	**-**	-	-	**-**
Yes	240(83.0)	49(17.0)	289	**<0.001**
No	1433(53.2)	1,258(46.8)	2,691	**-**
**Have you ever given birth?**	**-**			-
Yes	446(35.2)	822(64.8)	1,268	**<0.001**
No or pregnant now	187(76.0)	59(24.0)	246	-
**Number of children since 2012**	-			-
None	61(43.9)	78(56.1)	139	0.071
1 child	383(34.1)	739(65.9)	1,122	-
2 to 4 children	2(28.6)	5(71.9)	7	-

**Table 3:** factors associated with having risky sexual behaviors among AGYW who reported ever having sex, population-based HIV impact assessment in Rwanda (October 2018 to March 2019)

Participants’ characteristics	aOR*	95% CI Lower Upper		p-value
**Sociodemographic characteristics**	-	-	-	-
Age	-	-	-	-
15-19 years	Ref**	-	-	-
20-24 years	0.7	0.5	1.1	0.093
**Ever been married to or lived with a man**				-
Yes	Ref			-
No	0.8	0.6	1.0	0.088
**Household characteristics**	-	-	-	-
**The number of children in the household**	-	-	-	-
No child	Ref	-	-	-
1 to 3	1.9	1.3	2.7	0.001
4 to 6	1.6	1.0	2.5	0.037
7 to 11	1.9	0.8	4.2	0.131
**Sexual and obstetric characteristics**	-	-	-	-
**Age at first sex**	-	-	-	-
Below 15	Omitted	-	-	-
15-17	1.3	1.0	1.7	0.019
18-24	Ref	-	-	-
**Sex before the age of 15**	-	-	-	-
Yes	1.8	1.1	2.9	0.017
No	Ref	-	-	-
**Engaged in a sexual relationship with a person she resides with**	-	-	-	-
No	0.9	0.7	1.2	0.613
Yes	Ref	-		
**Whether ever been pregnant**	-	-		
No	4.3	1.4	13.4	0.011
Yes	Ref	-	-	-
**Whether currently pregnant**		-	-	-
Yes	Ref	-	-	-
No	9.5	6.8	13.4	<0.001
**Whether ever had a pregnancy that resulted in a live birth**	-	-		
Yes	Ref	-	-	-
No or pregnant now	0.8	0.5	1.2	0.297

* aOR: adjusted odds ratio - Predicted probabilities indicate membership in having risky sexual behaviors. **Ref: Reference group equivalent to 1

All variables in Table 2 and Table 2.2 with a p-value below 0.05 were included in a jackknife logistic regression model to produce adjusted odds ratios. The findings indicate that AGYW who were not pregnant and had never been pregnant by the time of the interview were 9.5 times (aOR: 9.5, 95% C.I: 6.8-13.4) and 4.3 times (aOR: 4.3, 95% C.I: 1.4-13.4) more likely to present RSBs than those who were pregnant. Similarly, slightly higher odds ratios were observed among AGYW living in a household with several children ranging from 1 to 3 (aOR: 1.9 with 95% C.I: 1.3-2.7) and 4 to 6 (aOR: 1.6 with 95% C.I: 1.0-2.5), who started sex before the age of 15 (aOR: 1.8 with 95% C.I: 1.1-2.9), and who had their sex debut at an age between 15 and 17 years (aOR: 1.3 with 95% C.I: 1.0-1.7). Detailed information is found in Table 3.

## Discussion

This study contributes to the scientific understanding of the prevalence and types of risky sexual behaviors (RSBs) among AGYW, as well as the factors influencing these behaviors in populations disproportionately at risk of HIV. It is the first study to document RSBs among AGYW in Rwanda using population-based data from the Rwanda HIV Impact Assessment (PHIA) survey. The findings are discussed in the context of recommended practices and compared with results from similar studies conducted globally, regionally, and nationally. Explanations for the observed patterns are also provided.

**The prevalence of risky sexual behaviors among AGYW:** this study shows that of the 2980 AGYW who reported ever having sex in the Rwanda PHIA, 43.9% engaged in risky sexual behaviors. This prevalence is higher than 43.1% [15] reported in Ethiopia and 41% previously reported in Rwanda [16], but lower than 53.9% in Ethiopia [17], 69.5% in Thailand [18], and 71.1% in Zambia among female adolescents [8]. Differences can be attributed to methodological approaches and geographic distributions of the study participants. In Rwanda, these findings reflect the economic vulnerability of young people and their dependence on men, as well as cultural or social norms that may limit their ability to negotiate safe sex [2,12]. Despite the Rwandan government's law punishing anyone engaging in sexual intercourse with children under 18 years of age [19] and efforts to equip youths with SRH knowledge through its partners, these results may be a proxy for sexual violence towards AGYW.

**Types of risky sexual behaviors identified among AGYW who reported ever having sex:** the findings show that the most common RSBs among AGYW include having multiple sexual partners (9.1%), not using condoms (13.5%) during extra-marital sex in the last 12 months, and having sex without a reliable method of contraception (24.8%). This study shows that 44.1% of the study participants have ever had sex, and most of them have had it with multiple sexual partners (9.1%), while about 5.1% had sex before the age of 15. These findings differ from the findings in other studies, such as a Ugandan study [20]. The Ugandan study reported relatively higher rates of AGYW having ever had sex (54.5%) and sex before age 15 (19.1%), but the current study reported a much lower prevalence of AGYW with multiple sexual partners (9.1%) compared to 24.2% in Uganda [20]. Several other studies reported lower rates of having two or more partners, such as 66.2% in Colombia [21] , 59.4% in Mozambique [22], 47% in Moshi SS, Tanzania [9], 45% in Ethiopia [7], and 20.4% in Ethiopia [7]. Variations can be attributed to disparities in methodological approaches in the surveys.

Sex without a condom was also very common among AGYW who reported ever having sex in Rwanda (86.5%). The findings are comparable to those reported in other countries, including 79.6% in Uganda [20], 74% in Kenya [10], and 61.2% in Zambia [23]. However, the rate is much higher than the 73% reported in Colombia [21], 22.7% in Ethiopia [7] and 40.6% in South Africa [24]. Differences can be due to the study populations enrolled in these studies and community sensitisation on the use of youth-friendly sexual and reproductive health services in these countries. The majority of AGYW who reported ever having had sex were not using any methods of contraception (75.2%). This prevalence is disproportionately higher than that reported in Kenya at 37.4% [25], in South Africa at 47.7% (1% sterilized, 9.6% pill users, 2.3% intrauterine device, and 34.8% on injectable) [24], and in Mozambique at 42.0% [22]. Moreover, a Kenyan study revealed that AGYW in universities are more worried about getting pregnant than contracting sexually transmitted infections, including HIV. However, instead of using long-acting contraception, they merely rely on emergency contraception methods [26]. This calls for action to increase AGYW awareness to consider STI prevention equally, along with pregnancy planning. Although sex for gifts/favor/money (9.9%), transgenerational sex (1.7%), and anal sex (0.8%) were relatively uncommon among AGYW aged 15 to 24 years in Rwanda, several other studies have reported higher rates of these RSBs in South Africa [27], including 14% of transactional sex in South Africa [28], along with many others [27,28].

**Factors associated with engaging in risky sexual behaviors among AGYW who reported ever having sex:** overall, the findings indicate that among all the participants' characteristics analyzed, such as age, province of residence, marital history, media exposure, and number of children, only three characteristics predicted risky sexual behaviors among AGYW. The analysis shows that significant predictors of RSBs among AGYW include a lack of pregnancy history, not being pregnant at the time of the interview, living with more children, and a lower age of sexual debut.

Firstly, AGYW who reported not being pregnant at the time of the study or having never been pregnant were more likely to engage in RSBs compared to their counterparts who had experienced pregnancy. This finding can be attributed to increased awareness and perceived risks of unintended pregnancies and STIs among AGYW who are pregnant or have previously been pregnant.

In this context, pregnancy among AGYW often prompts counselling and education on sexual and reproductive health from peers, parents, and healthcare providers. This exposure enhances their knowledge and encourages the adoption of methods to prevent both pregnancies and STIs, including HIV/AIDS. Additionally, experiencing pregnancy serves as a powerful reminder of how easily one can become pregnant or contract an STI. This heightened awareness likely increases their perceived susceptibility to the consequences of RSBs.

As a result, pregnancy can lead to improved self-perception, empowerment, and access to support systems, enabling AGYW to undergo psychological shifts that promote caution and prioritize stability. Furthermore, AGYW with children may benefit from stronger family or partner support systems, which can discourage RSBs. In contrast, AGYW without children may have greater freedom and fewer perceived constraints, potentially leading to higher engagement in riskier behaviors. Secondly, living with an increasing number of children in a household is highly associated with risky sexual behaviors among AGYW. This may be attributed to the possibility that parents or guardians are unable to monitor the increasing number of children and fulfil the financial needs of larger families. It can also serve as a proxy for a lack of role models and peer pressure.

Thirdly, a lower age of sexual debut and sex before the age of 15 years were predictors of risky sexual behaviors among the AGYW. This may be a result of normalising risky sexual behaviors like having multiple sexual partners, inconsistent or no use of condoms, and engaging in transactional sex since their first sex at a younger age. Similarly, these findings underscore the lifelong and detrimental effects of gender-based violence. Sex before the age of 15 has been reported to be at the root of lifelong sexual and reproductive health problems. Particularly in Rwanda, previous studies have reported an increased risk of engaging in sex work and RSBs among females who start sex at a younger age, such as before 15 years [24]. A history of sexual child abuse was also found to predict RSBs in Thailand (AOR = 1.60) [20], along with domestic violence (aOR: 4.2) in Rwanda [25]. Similar findings were reported in Mozambique, where early sexual debut was associated with RSBs (AOR: 1.37) [22]. In response to this risk, interventions to delay the age of sexual debut would also improve the overall health of young females.

Compared with previous studies, the findings of this study are in line with several other studies, especially those in low and middle-income countries. However, additional factors were also identified as predictors of RSBs among AGYW. The factors include a history of alcohol consumption and smoking, having a sexually active colleague in Ethiopia [15], not being on good terms with one's mother [29], experiencing domestic violence, living away from biological children in Rwanda [16], being raised by a single parent or grandparent, using dating applications in China [30], being between the ages of 20 to 23 years, and having inadequate knowledge about HIV/AIDS in Ethiopia [15].

From a public health perspective, these findings highlight the need for targeted sexual and reproductive health interventions to reduce risky sexual behaviors among AGYW. Interventions that promote delayed sexual debut, consistent use of contraception and condoms, and address underlying social and economic vulnerabilities may improve sexual health outcomes and reduce the risk of unintended pregnancies and sexually transmitted infections, including HIV. In addition, engaging AGYW with prior pregnancy experience as peer educators may enhance the effectiveness of peer-led prevention efforts. Although the study was conducted in Rwanda, the findings are likely applicable to similar low- and middle-income settings where AGYW face comparable sexual and reproductive health risks.

**Data availability:** the data that support the findings of this study are available from ICAP at the Columbia University website (https://phia-data.icap.columbia.edu/datasets?country_id=11). However, the cleaned and processed data (STATA DTA and Do-file) can be obtained from the corresponding author upon request.

**Limitations of the study:** this study focused on risky sexual behaviors among young females aged 15 to 24 years who were enrolled in the Population-based HIV Impact Assessment Survey in Rwanda from Oct 2018 to March 2019. Although the current study yielded important findings on factors associated with RSBs among AGYW, it did not establish a causal relationship between the risky sexual behaviors and identified factors. Moreover, this study used secondary data, and therefore, it relied on information available in the dataset.

## Conclusion

Overall, this study found that nearly half (43.9%) of AGYW aged 15-24 who had ever had sex engaged in at least one risky sexual behavior. The most common behaviors included sex without a reliable contraceptive method (24.8%), extramarital sex without a condom (13.5%), early sexual debut before age 15 (11.5%), transactional sex (9.9%), multiple sexual partners (9.1%), transgenerational sex (1.7%), and anal sex (0.8%). Risky sexual behaviors were strongly associated with being pregnant at the time of the interview (aOR: 9.5, 95% CI: 6.8-13.4), having a prior pregnancy (aOR: 4.3, 95% CI: 1.4-13.4), early sexual debut before age 15 (aOR: 1.8, 95% CI: 1.1-2.9), first sex at 15-17 years (aOR: 1.3, 95% CI: 1.0-1.7), and living in households with children under 15 years (aORs: 1.6-1.9). These findings suggest that sexual and reproductive health programs should prioritise delaying sexual debut, promoting contraceptive use, and supporting family planning in households to reduce risky sexual behaviors among AGYW.

### What is known about this topic

- RSBs are influenced by various demographic, socio-economic, and cultural factors in Africa;
- In Rwanda, adolescent girls and young women are at a heightened risk of HIV infection, STIs, and unintended pregnancies due to engagement in RSBs;
- However, the prevalence and factors driving these behaviors were not yet studied in the Rwandan context.

### What this study adds

- High population-level burden of risky sexual behaviors: nearly half (43.9%) of sexually active adolescent girls and young women (AGYW) in Rwanda engage in at least one risky sexual behavior, providing the first weighted national estimate for this population;
- Dominance of contraception- and condom-related risks; the most prevalent risky sexual behaviors are sex without a reliable contraceptive method and condomless extramarital sex, indicating that sexual risk among AGYW is primarily driven by inadequate protection rather than multiple partnerships or transactional sex;
- Pregnancy and early sexual debut as key determinants: current or prior pregnancy and early initiation of sexual activity (before age 18, particularly before age 15) emerge as the strongest predictors of risky sexual behaviors, highlighting sustained vulnerability among these subgroups.
